# A predicted physicochemically distinct sub-proteome associated with the intracellular organelle of the anammox bacterium *Kuenenia stuttgartiensis*

**DOI:** 10.1186/1471-2164-11-299

**Published:** 2010-05-12

**Authors:** Marnix H Medema, Miaomiao Zhou, Sacha AFT van Hijum, Jolein Gloerich, Hans JCT Wessels, Roland J Siezen, Marc Strous

**Affiliations:** 1 Department of Microbiology, Radboud University Nijmegen, Toernooiveld 1, 6525 ED Nijmegen, the Netherlands; 2 Centre for Molecular and Biomolecular Informatics, Radboud University Nijmegen Medical Centre, PO Box 9101, 6500 HB Nijmegen, the Netherlands; 3 NIZO food research, PO Box 20, 6710 BA Ede, the Netherlands; 4 Nijmegen Proteomics Facility, Laboratory of Pediatrics & Neurology, Radboud University Nijmegen Medical Centre, Nijmegen, the Netherlands; 5 TI Food and Nutrition, Kluyver Centre for Genomics of Industrial Fermentation, Wageningen, the Netherlands; 6 MPI for Marine Microbiology, Celsiusstr. 1 D-28359, Bremen, Germany; 7 CeBiTec, University of Bielefeld, Universitätsstraße 27, D-33615, Bielefeld, Germany; 8 Current Address: Department of Microbial Physiology and Groningen Bioinformatics Centre, Groningen Biomolecular Sciences and Biotechnology Institute, University of Groningen, Kerklaan 30, 9751 NN Haren, the Netherlands

## Abstract

**Background:**

Anaerobic ammonium-oxidizing (anammox) bacteria perform a key step in global nitrogen cycling. These bacteria make use of an organelle to oxidize ammonia anaerobically to nitrogen (N_2_) and so contribute ~50% of the nitrogen in the atmosphere. It is currently unknown which proteins constitute the organellar proteome and how anammox bacteria are able to specifically target organellar and cell-envelope proteins to their correct final destinations. Experimental approaches are complicated by the absence of pure cultures and genetic accessibility. However, the genome of the anammox bacterium Candidatus "*Kuenenia stuttgartiensis" *has recently been sequenced. Here, we make use of these genome data to predict the organellar sub-proteome and address the molecular basis of protein sorting in anammox bacteria.

**Results:**

Two training sets representing organellar (30 proteins) and cell envelope (59 proteins) proteins were constructed based on previous experimental evidence and comparative genomics. Random forest (RF) classifiers trained on these two sets could differentiate between organellar and cell envelope proteins with ~89% accuracy using 400 features consisting of frequencies of two adjacent amino acid combinations. A physicochemically distinct organellar sub-proteome containing 562 proteins was predicted with the best RF classifier. This set included almost all catabolic and respiratory factors encoded in the genome. Apparently, the cytoplasmic membrane performs no catabolic functions. We predict that the Tat-translocation system is located exclusively in the organellar membrane, whereas the Sec-translocation system is located on both the organellar and cytoplasmic membranes. Canonical signal peptides were predicted and validated experimentally, but a specific (N- or C-terminal) signal that could be used for protein targeting to the organelle remained elusive.

**Conclusions:**

A physicochemically distinct organellar sub-proteome was predicted from the genome of the anammox bacterium *K. stuttgartiensis*. This result provides strong *in silico *support for the existing experimental evidence for the existence of an organelle in this bacterium, and is an important step forward in unravelling a geochemically relevant case of cytoplasmic differentiation in bacteria. The predicted dual location of the Sec-translocation system and the apparent absence of a specific N- or C-terminal signal in the organellar proteins suggests that additional chaperones may be necessary that act on an as-yet unknown property of the targeted proteins.

## Background

Anaerobic ammonium-oxidizing (anammox) bacteria convert ammonium and nitrite into nitrogen and are major players in the biogeochemical nitrogen cycle [1-4]. They comprise a monophyletic taxon within the *Planctomycetes *phylum. Like other *Planctomycetes*, they possess an unusual cellular architecture with a diderm cell envelope and a compartmentalized cytoplasm [5,6]. More specifically, the cells of anammox bacteria contain a single organelle-like intracytoplasmic compartment bounded by a single bilayer membrane. This compartment is known as the anammoxosome, and was proposed to be the site at which the anammox reaction takes place [7]. This reaction is thought to be performed mainly by cytochrome c enzymes [8]. Within anammox cells, such enzymes have been shown to be present exclusively inside the anammoxosome [9,10].

If indeed the anammoxosome is a separate compartment in which a distinct and substantial part of the proteome is localized, this would present a situation unique to bacteria. In a thorough electron tomographical study it was reported that, unlike for example the magnetosomes of magnetotactic bacteria [11] and the chlorosomes of green photosynthetic bacteria [12], the anammoxosome has no detectable membrane links with the cell envelope during its biogenesis [13]. Furthermore, anammoxosomes divide separately from the cell envelope during cell division [14].

This leads to two questions regarding the cell biology of anammox bacteria: Firstly, which proteins are targeted to the anammoxosome besides the cytochrome c enzymes? Secondly, by what mechanism are these proteins specifically targeted to the anammoxosome?

One possible answer to the second question is that anammoxosomal proteins might contain specific sorting signals such as targeting motifs, domains or signal peptides [15-19]. For example, in *Salmonella*, several effectors were reported to contain multifunctional motifs or domains that are responsible for translocation and localization of the effector traits [20]. Moreover, some cases have recently been discovered in which modulation of Sec-signal peptide sequences result in different protein localizations [21,22]. Most strikingly, in cyanobacteria, signal peptides from proteins targeted to the thylakoid differ from signal peptides of proteins targeted to the cell envelope [23-25].

Progress in the experimental investigation of the cell biology of anammox bacteria is slow because these bacteria grow exceptionally slowly (with a doubling time of two weeks), and are not available in pure culture. However, the genome of the anammox bacterium Candidatus *"Kuenenia stuttgartiensis" *was recently assembled from a community genome [8].

Using these genome sequence data, it might be possible to answer the first question. Interestingly, it has been shown experimentally that the anammoxosome may be more acidic than both the cytoplasm and the cell envelope [26]. We reasoned that such a physicochemical difference could be reflected in the amino acid composition of the anammoxosomal sub-proteome [27], and that this difference could be used to predict this sub-proteome *in silico*. Therefore, a Random forest (RF) classifier was trained on two sets of anammoxosomal (set A) and cell-envelope (set P) protein sequences, constructed based on existing experimental evidence and comparative genomics. The best RF classifier was successful at predicting the targeting of proteins to the anammoxosome. This approach was complemented by the analysis of the encoded protein translocation machinery. Finally, the predicted signal peptides of the two sub-proteomes were analyzed and compared to unravel the molecular basis of protein sorting in anammox bacteria.

## Results

### Construction of training sets

We constructed two sets of amino acid sequences from soluble proteins with functions that were known to be specific to either the anammoxosome or the cell envelope (Additional file 1: sheet S1). The anammoxosomal set (termed 'set A') contained the sequences of 30 proteins, including the 26 most highly expressed cytochrome c proteins of *K. stuttgartiensis *[8,10] and 4 orthologues of these proteins from another anammox bacterium, Candidatus "*Scalindua marina" *(data kindly provided by M. Jetten and M. Kuypers). The cell-envelope set (termed 'set P') contained the sequences of 59 proteins that were homologous to proteins with an experimentally validated function specific for the periplasm, cell envelope, or extracellular environment. All proteins of both sets contained a predicted N-terminal signal peptide that can putatively be recognized by the Sec translocon. TatFind [28] and PilFind [29] predicted no Tat-system or Type IV system-secreted proteins in these two sets.

### Training of the Random forest classifier

To detect the overall compositional differences between the anammoxosomal and cell-envelope protein sets, we constructed Random forest (RF) classifiers based on amino acid composition, using set A and set P as the training sets. Among commonly used analytical machine-learning techniques [30-35], the RF algorithm has shown its power in classifying proteins based on noisy amino acid composition [36-40]. To balance class sizes of sets A and P, set P was first randomized into two sets (P1 and P2) to arrive at three equally sized protein sets (see Material and Methods).

**Figure 1:**
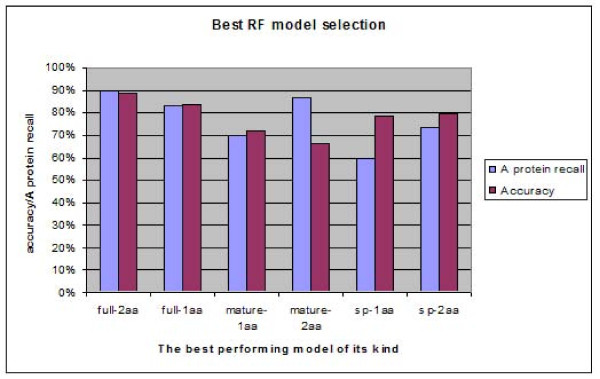
**Performance comparison of the RF model trained on different types of input data**. 500 RF models with randomly generated P1 and P2 sets, to correct for class A and P inbalance, were trained on each of the following 6 types of data: the full-length amino acid sequences, the signal peptides (SP) and the mature protein amino acid sequences, each analyzed with either the residue frequency of single amino acids or the frequency of 2 adjacent amino acids. When the 6 top-performing models of each input type are compared, the model trained with full-length protein sequences with the 2 adjacent amino acids combination shows the highest overall accuracy (89%) and A protein recall (90%).

In total 3000 three-set (A, P1, P2) RF classifiers were trained, based on different combinations of single or two adjacent amino acids frequencies (average out-of-bag (OOB) error 24.4% with standard deviation of 9.7%). The best-performing RF model for discriminating set A from set P was selected based on the highest accuracy (89%) and the best anammoxosomal protein (set A) recall (90%). This RF model had been made using two-adjacent-amino-acids combination frequencies from full-length protein sequences as the input (Figure 1). The top 10 most important adjacent amino acid residue combinations associated with the recognition of set A were GP, TS, ID, YS, TF, LD, YG, IG, GN and IT. The highest accuracy of RFs trained on other types of input data (with single amino acid frequencies or with other regions of the protein sequences, e.g. signal peptides) ranged from ~75% to 84%, and the recall of anammoxosomal protein sequences ranged from 60% to 83%, respectively (Figure 1). No tests on combinations of more than two amino acids were attempted because this required prohibitively long computation time. During the training process, the RF algorithm chose the most representative features of each class by random bagging with overlaps, which is similar to jack-knife cross validations. Within the training process, RF accurately determines classification error-rate (out-of-bag errors; OOB error) [40-42]. These OOB errors were used as a non-biased indicator of the performance of the classifiers.

### Identification of translocated and membrane proteins

**Figure 2:**
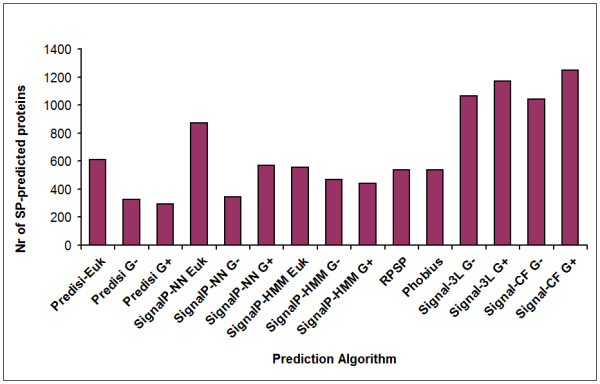
**Signal peptide predictions on the whole proteome of *K. stuttgartiensis***. Signal peptide predictions on the whole proteome (4663 proteins) of *K. stuttgartiensis *by fifteen signal peptide prediction algorithms. The *y*-axis shows the number of proteins predicted to carry a signal peptide. Abbreviations: G+: predict option of Gram-positive; G-: predict option of Gram-negative; Euk: predict option of Eukaryote. The number of predicted Tat and Type IV prepilin substrates using TatFind and PilFind were nine and ten, respectively.

All translocated proteins and membrane proteins encoded in the genome of *K. stuttgartiensis *were classified based on the predicted presence of transmembrane helices and/or signal peptides. Prediction of signal peptides was not straightforward because anammox bacteria are evolutionarily only distantly related to proteins of those organisms that were used to train the predictors (e.g. *Proteobacteria *or Gram-positive bacteria). For this reason, 15 different available signal-peptide prediction algorithms were applied to the open-reading frames predicted for the *K. stuttgartiensis *genome (Figure 2). Positive predictions were combined into a single majority vote decision for each protein. Among 4663 open-reading frames, 594 membrane proteins and 344 translocated soluble proteins with signal peptides were predicted (Additional file 1: sheet S2). Nine of the signal-peptide carrying proteins were predicted to be Tat-translocated by TatFind, and ten proteins were predicted to be secreted by the Type IV secretion system by PilFind.

**Figure 3:**
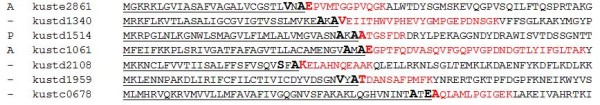
**Experimental validation of signal peptides in the Candidatus *Kuenenia stuttgartiensis *proteins**. Identification of cleavage sites from seven Candidatus Kuenenia stuttgartiensis mature proteins. The peptides that were identified in the tryptic digest are coloured red; their N-terminal sides were non-tryptic. Underlined sequences represent the putative signal peptides, and the putative SPase 1 recognition sites adjacent to the non-tryptic side of the peptides are printed in bold. The left column indicates whether the protein is present in either the A or the P training set.

Experimental evidence was obtained to confirm the signal peptide cleavage sites of some exemplary proteins by protein mass-spectrometry. For seven K. stuttgartiensis mature proteins, including two from set A and one from set P, N-terminally non-tryptic peptides were identified that matched exactly to sequences after a putative SP1-cleavage site at the end of a canonical Sec signal peptide (Figure 3), indicating that the signal peptides of both anammoxosomal and cell-envelope proteins were predicted correctly and are functional.

### Prediction of the anammoxosomal sub-proteome

We then used our RF classifier to predict the destination of the 938 translocated and membrane proteins. Of these proteins, approximately 60% (562 proteins) was predicted to be anammoxosomal (Table 1, and Additional file 2: sheet S2) after removal of four predicted type IV secretion system substrates.

**Table 1:** Composition of the predicted organellar proteome of K. stuttgartiensis.

Protein family	Examples of the predicted organellar proteins	Locus tag	# proteins
Cytochrome C proteins	cytochrome c551 peroxidase	kuste2905	49
	cytochrome c6	kustc0563	
	cbb3-type cytochrome c oxidase subunit 1 (CcoN)	kustc0429	
	hepta heme protein	kuste2855	

Respiratory complex proteins	cd1 nitrite reductase (NirS)	kuste4136	50
	NAD(P)H:quinone oxidoreductase chain 5	kustc0838	
	proton-translocating NADH dehydrogenase I chain A (NuoA)	kustc0822	
	NADH:ubiquinone oxidoreductase subunit M	kustc0840	

Transporters	Ammonium transporter 1	kustc0381	27
	Nitrite Transporter 1 (FocA)	kustd1720	
	Nitrite/nitrate antiporter (NarK)	kuste2335	
	copper-transporting ATPase	kuste2247	

Protein translocation system	SecDF-YajC accessory complex (YajC)	kustd1963	5
	SecYEG translocation complex (SecE)	kuste2951	
	Transmembrane pore (TatC)	kustc0286	
	Tat signal recognition (TatA/B)	kuste2348	

Cytochrome C maturation system	thiol-disulfide oxidoreductase (ResA)	kustc0860	6
	cytochrome c-type biogenesis protein (ResC)	kustd1760	
	thiol:disulfide interchange protein (DsbD)	kustc0946	

TPR proteins	N-acetylglucosaminyltransferase (O-GlcNAc transferase)	kuste2787	32
	kinesin light chain KLC (putative)	kuste2807	

Hypothetical and other proteins			392

Total proteins			562

A summary of the predicted anammoxosomal sub-proteome of *K. stuttgartiensis*. In this set, 371 proteins are hypothetical proteins of unknown function. Some examples of proteins are listed, especially those with functions known to be related to anammox. Details of the predicted sub-proteome are stored in Additional file 2.

Importantly, the predicted anammoxosomal sub-proteome formed a functionally consistent and cohesive set. First, the cytochrome c maturation machinery [43] was predicted to be anammoxosomal as 6 out of the 8 encoding genes were predicted to be targeted to the anammoxosome (Additional file 2: sheet S4), consistent with the recent proteomics study of Karlsson *et al*. [9]. Secondly, the destination of proteins encoded in the same genetic neighbourhood (i.e. putative operons) was generally consistent. This makes sense because such proteins are usually subunits of a protein complex or otherwise functionally associated (Additional file 2: sheet S5). Thirdly, we found that only 15 of the 562 predicted anammoxosomal proteins (2.7%) had SMART or PFAM functional domains [44] (e-value < 0.01) which are on functional grounds incompatible with an anammoxosomal location (Additional file 2: sheet S2).

Finally, the predicted anammoxosomal sub-proteome was consistent with the proposed biological role of the anammoxosome [7,10,45]. If the main catabolism of anammox bacteria takes place in the anammoxosome, the respiratory complexes should be associated with this compartment. Indeed, all 14 major respiratory complexes encoded in the *K. stuttgartiensis *genome were predicted to reside in the anammoxosomal membrane (Additional file 2: sheet S6). Moreover, three out of four ammonium transporters, all nitrite transporters, and all nitrite/nitrate antiporters were predicted to be anammoxosomal (Additional file 2: sheet S7). In contrast, importers of essential trace elements and amino acids, as well as multidrug-efflux proteins were predicted to be located on the outside of the cells, as expected. A single putative copper ATP transporter was predicted to be anammoxosomal consistent with the anammoxosomal destination of some enzymes dependent on copper (or other metal cations).

Overall, the RF classifier predicts that in anammox bacteria the cytoplasmic membrane is mainly used for transport and that essentially all catabolic functions (the anammox reaction, respiration and ATP synthesis) are associated with the intracytoplasmic organelle.

### Mechanism of protein translocation

The next point we addressed is the molecular basis for protein sorting in anammox bacteria. We reasoned that comparison of predicted protein features to those of reference bacteria could provide the first clues to how such a sorting system could function.

Homology searches showed that the typical bacterial protein translocation system components, including the Sec-translocation system (SecYEG, SecA and YidC proteins) [46-48], Tat-translocation system (TatA/B and TatC proteins) [49] and type I [50], II [51] and IV [52] signal peptidases were encoded by the genome of *K. stuttgartiensis*. All components were present in single gene copy only (Table 2).

**Table 2:** Protein sorting components encoded in the K. stuttgartiensis genome

*Candidatus Kuenenia stuttgartiensis *homologues of proteins involved in protein sorting				
**Protein**	**Function**	**Subcellular Location**	***Kuenenia *homologue**	**Accession Number**

SecY	SecYEG translocation complex	Membrane	kuste2983	CAJ73737
SecE	SecYEG translocation complex	Membrane	kuste2951	CAJ73704
SecG	SecYEG translocation complex	Membrane	kuste4254	CAJ75016
SecB	chaperone	Cytoplasm	-	-
SecA	ATPase motor protein	Cytoplasm	kustb0170	CAJ70915
SecDF	SecDF-YajC accessory complex	Membrane	kustd1962	CAJ72707
YajC	SecDF-YajC accessory complex	Membrane	kustd1963	CAJ72708
YidC	membrane protein assembly	Membrane	kustd1734	CAJ72479

TatA/B	Transmembrane pore	Membrane	kuste2348	CAJ73093
TatC	Tat signal recognition	Membrane	kustc0286	CAJ71031

Signal Peptidase I	Sec signal peptidase	Membrane	kuste3749	CAJ74512
Signal Peptidase II	lipoprotein signal peptidase	Membrane	kuste4338	CAJ75100
Signal Peptidase IV	prepilin signal peptidase	Membrane	kustc0984	CAJ71729

FtsY	SRP receptor	Membrane	kustc0279	CAJ71024
Ffh	Major SRP subunit	Cytoplasm	kuste3317	CAJ74078

Putative Candidatus *Kuenenia stuttgartiensis *orthologues of proteins that are known to be involved in protein sorting. Orthology is based on reciprocal best Blast hits and on the unique presence of PFAM functional domains.

The identified components of the Sec- and Tat-systems appeared canonical, except for the presence of a C-terminal FecR domain (PF04773) in TatC. This signal-transducing domain is absent in any other TatC protein identified so far. Topology predictions of the TatC-FecR protein (by Phobius [53], TMHMM [54], and HMMTOP [55]) unanimously showed that the FecR domain is non-cytoplasmic. Because of the uniqueness of such a domain combination, it is tempting to speculate that the FecR-like domain may somehow have a role in the evolutionary solution that has been found by anammox bacteria for protein sorting to the anammoxosome.

Interestingly, the RF classifier results for Sec components were ambiguous (only 3 out of 6 subunits were predicted to be anammoxosomal), whereas the Tat system was predicted to be completely anammoxosomal (Additional file 2: sheet S8). Consistently, 7 out of the 9 Tat-substrates predicted in the *K. stuttgartiensis *genome were also predicted to be anammoxosomal. These include a multi-copper oxidase SufI (kuste4301), a putative superoxide dismutase (kustd1303), two Rieske subunits of the bc1 complex (kuste3096 and kuste4569), and a few hypothetical proteins. The NarG nitrate reductase subunit is very probably a false positive, as has been noted earlier [56].

In conclusion, the encoded protein translocation machinery shows that no duplication of the Sec- or Tat-systems has taken place to facilitate separate translocation routes, and that both systems may be involved in protein sorting towards the anammoxosome.

### The role of signal peptides in protein sorting

Regardless of the protein translocation machinery used, the targeting of proteins to specific subcellular locations is often accomplished by modulation of N- or C-terminal signal peptides, in eukaryotes [15-19] and prokaryotes [23-25] alike. Therefore, we compared the N- and C-termini of the two training sets A and P to identify a possible distinctive property or amino acid motif that could be used to differentiate both sets of protein sequences.

**Figure 4:**
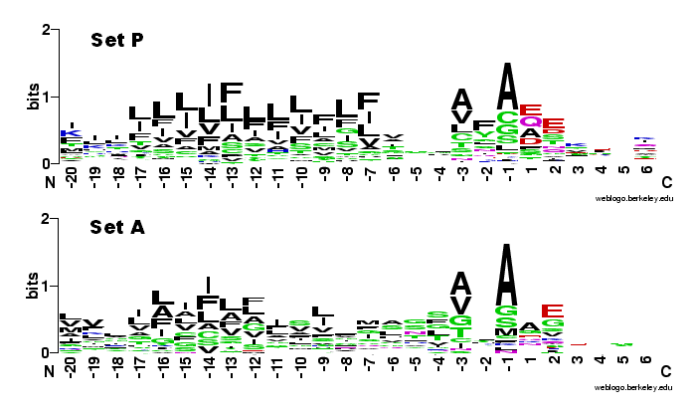
**Sequence composition of the signal peptides of the anammoxosome and cell envelope protein sets**. Weblogos of the signal peptides of protein sets A (anammoxosomal) and P (cell envelope) are shown. Both the hydrophobic h-regions (residues -6 to -17) and the signal peptidase AxA consensus (residues -1 to -3) preceding the cleavage site are clearly visible. The weblogos were created from sequences aligned to the cleavage site, using Weblogo [47].

Direct alignment of the signal peptides resulted in alignments which only had high quality (similarity level higher than 30%) in the h-regions (TMH). Therefore, the n-, h-, and c-regions of the signal peptides from the amino acid sequences from both training sets were first extracted manually, and were used to create a series of ungapped alignments which were aligned at: (1) the N-terminus; (2) the h-region start; (3) the c-region start; and (4) the putative cleavage site. The resulting segmental alignments were then joined in the order corresponding to the original signal peptide architecture. The sequence logo [57] of the ungapped alignments of the N-termini illustrates that in general the signal peptides of both sets were canonical type I signal peptides (Figure 4) and most of the proteins had SPase I cleavage sites with a clear AxA motif. These alignments were further investigated by comparing the search results of Hidden Markov Models [58] of both sets in a sliding window approach (Additional file 3), but no discriminating motif was found in either set A or set P. MEME motif searches [59] and statistical analysis of amino acid frequencies also did not yield any significant differences. Finally, we trained RF classifiers using only the N-terminus (30 amino acids) of the two sets of proteins, with the frequencies of two adjacent amino acid combinations as also used for the full protein RF classifiers. The resulting RF classifiers showed much lower accuracy than the models built based on the complete protein sequences, suggesting that less distinctive features were encoded in the N-terminus of these 2 groups of proteins (Figure 1).

An interesting side observation was that the h-regions of the predicted signal peptides from *K. stuttgartiensis *proteins contained significantly more phenylalanine residues (2.23 on average for both training sets) than found in *E. coli *TMHs (1.64 on average, Additional file 3: Supplemental Figure S3). We speculate that this difference may be related to the affinity of trans-membrane helices to the unique ladderane membranes of anammox bacteria that have an exceptionally high density to prevent diffusion [60].

Yet in conclusion, it is unlikely that the N- or C- terminus plays a role in protein targeting of anammox bacteria.

## Discussion

The anammoxosome of anammox bacteria is one of the best documented cases of organellar biogenesis in Bacteria; electron tomography has shown that this bacterial organelle divides separately from the cytoplasmic membrane, and is not connected to this membrane during a complete cell cycle [14]. Cytochrome c proteins were detected exclusively inside the anammoxosome [10] and an intracytoplasmic pH gradient was shown to exist [28]. Because experimental investigation of anammox bacteria appeared to be difficult and the genome of *K. stuttgartiensis *has been sequenced recently, an *in silico *analysis was therefore obviously the next step forwards in unravelling this interesting biological phenomenon.

**Figure 5:**
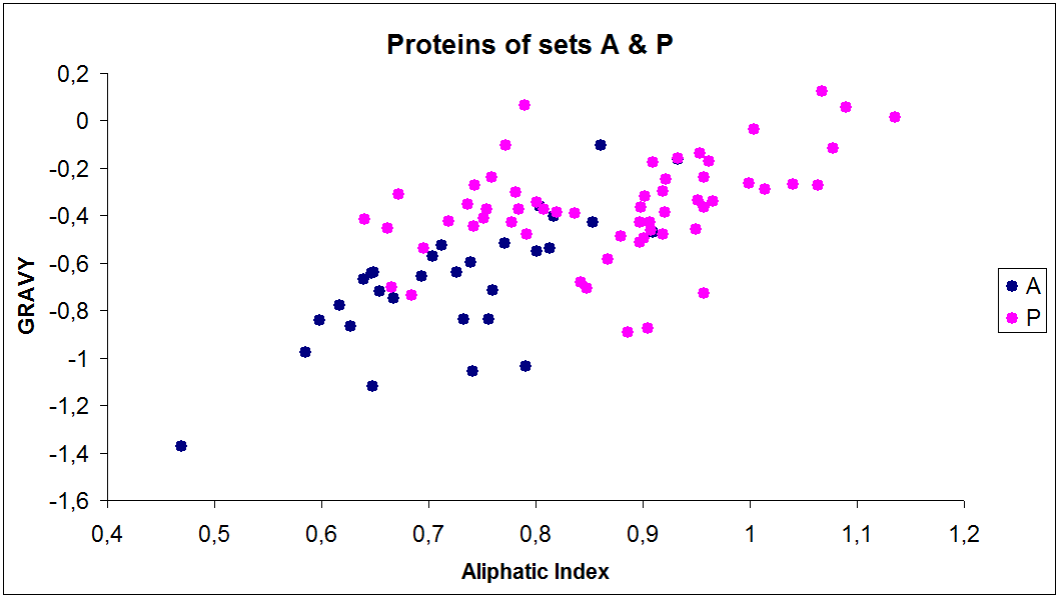
**Physicochemical differences between anammoxosomal and cell envelope proteins**. Two physicochemical parameters are plotted against each other: GRAVY index (grand average of hydropathy) and aliphatic index (relative volume occupied by aliphatic side chains of I, L, V, and A), which can both be calculated from amino acid compositions. These two parameters separate sets A and P into two largely distinct clusters. Purple dots: set P. Blue dots: set A.

In the present study a Random forest classifier was trained with two sets of protein sequences. The best RF classifier predicted an organellar sub-proteome of 562 proteins that was internally consistent and made functional sense. The best classifier used two-adjacent-amino-acids combination frequency as the input. According to their grand average of hydropathy (GRAVY) [61] and aliphatic index [62], the anammoxosomal proteins tend to be more hydropathic and more aliphatic (Figure 5). This could be a consequence of the different physico-chemical characteristics (e.g. different pH [26]) inside the anammoxosome. As computing power is likely to increase, the accuracy of the RF classifier may be improved in follow-up studies, e.g. by using combinations of more than 2 amino acids.

The anammoxosomal training set consisted only of amino acid sequences of cytochrome c proteins. It is possible that the classification was biased by the uniqueness of the protein types in this set. However, the cytochrome c protein family is only defined by the presence of a conserved CXXCH heme-binding motif (which we excluded from the RF input data) in an alpha-helical domain. Yet the remainder of the amino acid sequences were vastly variable, some polypeptides even containing regions with different folds or domains [63,64]. In fact, motif searches showed that no conserved sequence patterns could be found in set A except for the heme-binding motif, and the pairwise sequence identities of proteins in this set were all below 80%, with only 11 of them above 50%.

For the prediction of translocated proteins, a combination of 15 existing signal peptide prediction algorithms was used. These 15 signal peptide predictors reported immensely different predictions (Figure 2) on the presence or absence of a signal peptide in *K. stuttgartiensis *proteins. Considering the fact that the Gram-negative predictors were mainly trained with sequences from *Proteobacteria*, which are only very distantly related to anammox bacteria, it is highly unlikely that the reported accuracies of these predictors of 91-95% [65-68] can be valid for *Kuenenia *proteins. Moreover, among all employed algorithms, the SignalP-HMM algorithm trained on eukaryotes showed the highest true-positive rate by predicting signal peptides in 68 out of 69 of the proteins in set A. This indicates that anammox signal peptides are more similar to those of eukaryotes than to those of Gram-negative or Gram-positive model bacteria.

Analysis of the encoded protein translocation machinery provided some clues as to how this bacterium targets translocated proteins to their proper destination. Because this machinery is non-redundant, an additional layer of chaperoning would be required to achieve specificity. Moreover, because no sorting signal was apparent at the N- or C-termini of the proteins, such chaperones could act on the physicochemical characteristics observed in the amino acid sequence. Alternatively, the signal may act at the level of the messenger RNA that could determine the fate of the protein even before translation starts.

In case of the Sec translocase, some subunits (SecE, SecG and YidC) were predicted to be anammoxosomal, while some others (SecY, SecA, SecDF,YajC) were not. According to the presence of the seemingly canonical Sec-signal peptides on both cell-envelope-targeted proteins and anammoxosome-targeted proteins, it is likely that the Sec translocase has a dual localization on both the anammoxosomal and the periplasmic membranes. However, recent studies have also shown that under certain conditions Sec-system exported proteins could as well be translocated by the Tat-system [69,70], and therefore we cannot exclude the possibility that the Tat system translocates more than just the predicted twin-arginine-motif-carrying substrates.

An alternative hypothesis for anammoxosomal targeting could be primary translocation to the periplasm through the Sec pathway and secondary retro-translocation to the anammoxosome (or *vice versa*) through vesicles. However, it must be noted that the application of 3D electron microscopy (tomography) did not reveal any such vesicles in growing or dividing cells [13].

In order to further elucidate the anammox protein targeting problem, more experiments, such as immunolocalization of the Tat- and Sec-translocase subunits and quantitative proteomics approaches [71,72], comparing protein concentration levels in purified anammox cells and solutions enriched in anammoxosomes (a purification method which has been described earlier [60]), are required. The present study provides a clear hypothesis to future experiments: in anammox bacteria, catabolism and respiration are strictly organellar, leaving only transport functions for the cytoplasmic membrane.

## Conclusions

The anammoxosome of anammox bacteria is one of the best documented cases of organellar biogenesis in bacteria. Experiments have shown that several key enzymes catalyzing the anammox reaction are present exclusively inside the anammoxosome. The present study makes use of physicochemical characteristics of predicted protein sequences to predict a 90% accurate sub-proteome that constitutes this bacterial organelle. Meanwhile, the mechanism of protein sorting remained largely elusive.

The predicted sub-proteome has been deposited into a freely accessible Microsoft-Excel database (Additional file 2).

## Methods

### Genome sequence of Candidatus *K. stuttgartiensis*

The complete predicted proteome of *K. stuttgartiensis *was compiled from all annotated protein sequences (4663 ORFs, accessed 11-02-2008) encoded by *K. stuttgartiensis *genome fragments kustA - kustE (GenBank accession nrs.: CT030148, CT573074, CT573073, CT573072, CT573071). The proteome has been deposited in the peptidome database (accession number PSE111).

### Sets of putatively anammoxosome- and cell-envelope-targeted proteins

The set of amino acid sequences of putative anammoxosomal proteins ("set A") was first constructed from the cytochrome c proteins with a peptide coverage of more than 10% in an experimental analysis of the *K. stuttgartiensis *proteome (Kartal et al., unpublished data). Next, local BlastP searches were performed with the *Scalindu marina *metagenome using these protein sequences as queries in order to extend the set A. Reciprocal best Blast hits with the *K. stuttgartiensis *genome that share an identical gene context with the set A proteins were identified as orthologues and added to set A. By similar methods a set of amino acid sequences of putative cell-envelope and/or excreted proteins ("set P") was also constructed. This set consisted of proteins from the *K. stuttgartiensis *genome with high similarity to proteins with a validated function in the periplasm, cell envelope or extracellular environment [73-89].

Predicted integral transmembrane proteins (predicted using Phobius [53], combined with manual inspection) were removed from the sets. The translation start sites of the selected proteins were manually checked and corrected when necessary. Pairwise identities of the sequences from both sets were calculated with MatGAT [90] and redundant protein sequences (with pairwise identity higher than 85%) were removed.

### Prediction of signal peptide-carrying proteins in Candidatus *K. stuttgartiensis*

Signal peptide predictions on the whole *K. stuttgartiensis *proteome were performed by the Gram-negative, Gram-positive and eukaryote versions of the algorithm PrediSi [67], SignalP-HMM and SignalP-NN [66], Gram-negative and Gram-positive versions of Signal-3L [68] and Signal-CF [65], and the general versions of RPSP [91] and Phobius [53]. All positive predictions from all algorithms were combined into a majority vote decision. Trans-membrane helices (TMH) were predicted by Phobius, TMHMM [54], and HMMTOP [55]. Tat-secreted proteins were identified with TatFind [34]. The same algorithms were also run on all protein sequences of sets A and P (see below). The n-, h-, and c-regions of the predicted signal peptides were determined manually based on expert knowledge with help of the predictions by Phobius and SignalP. No attempt was made to differentiate between signal peptides (which are cleaved by a SPase) and signal anchors (which are not cleaved) for these sets.

### Random Forest classification based on mature protein amino acid composition

The Random Forest classifier package (version 4.5-28) [42,92] from the R environment (version 2.8.1) [42,93] was used to train RF classifiers for the separation of anammoxosomal (set A) and cell-envelope (set P) proteins.

In order to reduce the bias due to class size imbalance (larger size of set P), this set was randomly separated into two subsets (set P1 and P2) with sizes similar to that of set A. As input for the RF algorithm, features were determined based on the frequencies (occurrences divided by the sequence length) of amino acids, including the frequencies of two-adjacent-amino-acids, from (i) the SPs, (ii) the full-length amino acid sequences and (iii) the mature protein amino acid sequences of sets A, set P1 and set P2 (except cysteines and histidines, which constitute the heme c binding motif). A three-class (P1, P2, and A) RF model was trained with 1000 trees per forest using each set of input data at each round of P set randomization. The randomization training process was repeated 500 times for each set of input data, after which the votes for classes P1 and P2 were pooled into one merged set P and the overall classification or out-of-bag (OOB) errors were calculated.

The best RF model was selected based on overall accuracy and A protein recall. When a tie situation occurred with the overall accuracy, the model with higher A protein recall was preferred.

### Identification of Sec signal peptide cleavage sites by mass-spectrometry

Mass-spectrometry experiments were performed to identify the Sec-signal peptide cleavage sites in proteins from sets A and P. A detailed methodology of these experiments is described in the Additional file 3. The resulting mass spectrometric data files were searched against a database containing the *K. stuttgartiensis *proteins and known contaminants like human keratins and trypsin using Mascot (Matrix Science Inc., USA, version 2.2) [94]. Variant sequences were modified at the N-terminus by deleting amino acid 1 to 50 and were added to the database in order to search for the Sec-signal peptide cleavage site. The resulting peptide hits were validated using an in-house developed script which selects peptides based on peptide score, the number of variable modifications, the expectation value, and the modified delta score. From the list of validated peptides, a non-redundant N-terminal peptide list was manually extracted by the criteria of: (1), whether the protein was identified with ≥ 3 peptides; (2), whether the peptide was the first detectable peptide (based on calculated m/z values of *in silico *predicted tryptic peptides in relationship with the m/z detection limits of the mass spectrometer) of the protein; (3), whether the peptide was semi-tryptic (with the non-tryptic side at the N-terminus).

### Prediction of the anammoxosomal sub-proteome of *K. stuttgartiensis*

The Candidatus *K. stuttgartiensis *proteome was separated into the translocated and non-translocated sets by combining several signal-peptide predictors as follows: firstly, a majority vote of the prediction from 15 signal-peptide prediction algorithms (see above) was used to predict sets of proteins containing SPs (511) and proteins without SPs (4152). Secondly, the proteins without SPs were subdivided into sets of soluble cytoplasmic proteins (3724 proteins, set 4) and SP-less transmembrane proteins (427 proteins, set 3), using predictions of TMHMM 2.0 [54]. Finally the SP-containing proteins were considered subdivided into soluble proteins and proteins containing TMHs, by assessing which proteins had TMHs predicted by a Phobius constrained prediction (constraint: N-terminus = signal peptide). This resulted in a predicted set of 344 SP-containing soluble proteins (set 1) and a predicted set of 167 SP-containing transmembrane proteins (set 2) (Additional file 1: sheet S2). The TMHs (including TMH topology) were predicted by either a constrained Phobius search (for sets 1 and 2) or the TMHMM output (for set 3). Protein sequence composition data of subset 1-3 were then used to predict the anammoxosome proteome.

### Identification and sequence analysis of the protein translocation system components in the genome of Candidatus *Kuenenia stuttgartiensis*

Genes encoding translocation-associated proteins SecY, SecE, SecG, SecA, SecDF, YajC, YidC, TatA, TatC, SPI, SPII, SPIV, FtsY, and Ffh were identified in the *K. stuttgartiensis *genome by finding reciprocal best Blast hits using BlastP [95] with the well-studied proteins involved in inner membrane translocation in *Escherichia coli *K12 as queries. Orthologues from *S. marina *were identified by performing BlastP analysis on a metagenomic database (M. Jetten and M.Kuypers, unpublished results) constructed with a *S. marina *enrichment culture [96], using the above-identified *K. stuttgartiensis *protein sequences as a query.

Homologues of the Sec- and Tat- translocation system components from other bacterial species were found by PSI-Blast [97] searches using the *Escherichia coli K12 *proteins as queries on the GenBank http://www.ncbi.nlm.nih.gov/ genomic data from all bacterial phyla. The *K. stuttgartiensis *protein translocation-associated proteins were aligned to at least 4 non-planctomycete sequences using Muscle [98].

### Analysis of N-terminal signal peptides

Multiple sequence alignments were made by Muscle 3.6 using standard parameters. Motif searches were performed using MEME [59], first with the criterion of zero or one motif per sequence, then with one motif per sequence. Phylogenetic footprinting [99] was performed by aligning the signal peptides of *K. stuttgartiensis *and *S. marina *orthologues from set A together with their putative orthologues (based on reciprocal best BlastP hits) from the sequences of planctomycete KSU-1 obtained by Shimamura *et al*. [100,101]. The protein physicochemical properties, including the prevalence of general amino acid classes (ILV, FWY, AILVMFWYC, AGS, ST, GNP, DE, DN, KR, EQ, DENQ, HKR, DENQHKR, and DENQHKRST), aliphatic index [68], and GRAVY index (grand average of hydropathy, according to the Kyte/Doolittle scale) [61] of the proteins were calculated by custom Python scripts. Statistical analysis on these parameters was performed by calculating the average, standard deviation, and average deviation of the data.

## List of Abbreviations

HMM: hidden Markov model; AA: amino acid; RF: random forest; SP: signal peptide; TM: transmembrane; TMH: transmembrane helix; ORF: open reading frame; OOB: out-of-bag error estimate.

## Authors' contributions

MM carried out the sequence analysis and training set construction, performed biological interpretation of RF results and carried out *in silico *signal peptide analyses. MZ trained the Random Forest algorithm and performed the RF predictions. MM and MZ drafted the manuscript. SAFT advised in the Random Forest training. HW and JC carried out the proteomics analysis and supplied methods for the manuscript. MS initially conceived of the study. Both MS and RS participated in its coordination and helped to draft and finalize the manuscript. All authors read and approved the final manuscript.

## Supplementary Material

Additional file 1 **Experimental training sets**. The experimental training sets and the *K. stuttgartiensis *proteome subsets based on signal peptide predictions. Click here for file

Additional file 2 **Predicted anammoxosomal proteome**. Details on the anammoxosomal proteome prediction. Click here for file

Additional file 3 **Experimental and computational analysis of the signal peptides**. Methodological details of signal peptide mass spectrometry and analysis on signal peptides using motif searches, statistical analysis and HMMs. Supplemental figure S2 and S3 depict the signal peptide predictions by the 15 predictors on the training sets and weblogos of *K. stuttgartiensis *TMHs showing phenylalanine overrepresentation compared to *E. coli*. Click here for file
