# Supplementary material for: A predicted physicochemically distinct sub-proteome associated with the intracellular organelle of the anammox bacterium Kuenenia stuttgartiensis
Source: BMC Genomics. 2010 May 12;11:299. doi: 10.1186/1471-2164-11-299 (PMC2881027; doi:10.1186/1471-2164-11-299)
Supplement: Additional file 3 — Experimental and computational analysis of the signal peptides. Methodological details of signal peptide mass spectrometry and analysis on signal peptides using motif searches, statistical analysis and HMMs. Supplemental figure S2 and S3 depict the signal peptide predictions by the 15 predictors on the training sets and weblogos of K. stuttgartiensis TMHs showing phenylalanine overrepresentation compared to E. coli. [file 1471-2164-11-299-S3.DOC]

**Supplementary material:**

**Experimental and computational analysis of the signal peptides**

**Methods**

**Identification of Sec signal peptide cleavage sites by mass-spectrometry**

After polyacrylamide gel electrophoresis, the gels were stained with colloidal Coomassie as described elsewhere [1] . The gel lane was cut into 4 slices, and each gel slice was destained with three cycles of washing with successively 50 mM ammonium bicarbonate and 50% acetonitrile. Protein reduction, alkylation and digestion with trypsin were performed as previously described [2]. After digestion, the samples were desalted and purified [3]. Sample analysis by LC-MS/MS was performed using an Agilent nanoflow 1100 liquid chromatograph coupled on-line via a nano-electrospray ion source (Thermo Fisher Scientific) to a 7T linear ion-trap Fourier transform ion-cyclotron resonance mass spectrometer (LTQ FT, Thermo Fisher Scientific). The chromatographic column consisted of a 15 cm fused-silica emitter (New Objective, PicoTip Emitter, Tip: 8 +/- 1 µm, ID: 100 µm) packed with 3 μm C18 beads (Reprosil-Pur C18 AQ, Dr Maisch GMBH, Germany) [4]. After loading the peptides onto the column in buffer A (0.5% HAc), bound peptides were gradually eluted using a 67 minute gradient of buffer B (80% ACN, 0.5% HAc). First, the concentration of acetonitrile was increased from 2.4% to 8% in 5 minutes, followed by an increase from 8% to 24% acetonitrile in 55 minutes, and finally an increase from 24% to 40% acetonitrile in 7 minutes. The mass spectrometer was operated in positive ion mode and was programmed to analyze the top 4 most abundant ions from each precursor scan using dynamic exclusion. Survey mass spectra (350-2000 m/z) were recorded in the ICR cell at a resolution of R=5E5. Data dependent collision-induced fragmentation of the precursor ions was performed in the linear ion trap (normalized collision energy: 27%, activation q=0.250, activation time: 30ms).

Mass spectrometric data files were searched against a database containing the *K. stuttgartiensis* protein database and known contaminants like human keratins and trypsin. Database searches were performed using the database search program Mascot (Matrix Science Inc., USA, version 2.2)[5]. In order to obtain factors for the recalibration of precursor masses, initial searches were performed with a precursor ion tolerance of 50 ppm. Fragment ions were searched with 0.8 Da tolerance and searches allowed for 1 missed cleavage, carbamidomethylation (C) as fixed modification, and deamidation (NQ) and oxidation (M) as variable modifications. The results from these searches were used to calculate the m/z dependent deviation, which was used to recalibrate all precursor m/z values.

To search for putative signal peptides, the *K. stuttgartiensis* database was adapted: for each protein sequence in the database, 50 variant sequences were added. The variant sequences were modified at the N-terminus by deleting aa 1 to aa 1-50. To this modified *K. stuttgartiensis* database, known contaminants like human keratins and trypsin were added. The recalibrated precursor masses were used to perform a database search against the modified database. MASCOT database searches were performed using tryptic specificity with a maximum of 1 missed cleavage, a precursor ion tolerance of 20 ppm, and 0.8 Da tolerance for fragment ions. Carbamidomethylation (C) was set as fixed modification and variable modifications included oxidation (M), deamidation (NQ) and acetylation of protein N-termini. The resulting peptide hits were validated using an in-house developed script. This script selects peptides based on peptide score, the number of variable modifications, the expectation value, and the modified delta score. The modified delta score is the score between the first peptide match and the next peptide match with a different sequence. For proteins identified with more than 1 peptide, validation criteria for each peptide were: peptide score ≥ 30, a maximum of 3 variable modifications, an expectation value of ≤ 0.04, and a modified delta score of ≥ 10. For proteins identified by a single peptide the validation criteria were more strict: a peptide score of ≥ 50, a maximum of 1 variable modification, an expectation value of ≤ 0.03, and a modified delta score of ≥ 10.

From the list of validated peptides, a non-redundant peptide list was extracted containing only N-terminal peptides from proteins that were identified with ≥ 3 peptides. Of these peptides it was assessed manually whether the peptide was the first detectable peptide (based on calculated m/z values of *in* silico predicted tryptic peptides in relationship with the m/z detection limits of the mass spectrometer) of the protein, and whether the peptide was semi-tryptic (with the non-tryptic side at the N-terminus).

**Hidden Markov Model analysis of the signal peptides and C-termini sequence compositions**

Five ungapped alignments were constructed with the signal peptides and C-termini of protein sets A and P. These alignments contained: (1) the N-terminus, (2) four residues downstream of the start of the h-region, (3) fifteen residues downstream from the start of the c-region, (4) 20 residues downstream of the predicted signal peptide cleavage site, and (5) 30 residues from the C-terminus of the protein. For each of these ten alignments, sliding windows (sizes 5-25 residues) were taken from positions 1-30 of the alignments. For each of these windows, a Hidden Markov Model (HMM) was constructed with HMMer [6]. The built HMMs were applied to the corresponding sequence windows of both sets. HMM score distributions were constructed with distribution bins of 2 HMM score units. For every sequence window, the percentage of separation between the score distributions of the two sets for a given sequence window was calculated. Then, for every alignment the most discriminating region was determined by comparing the separation percentages and the central positions of the sliding windows for each window size. The most distinctive pair of HMMs was selected to separate the two sets. The highest and lowest HMM scores of each HMM were extracted as a first cut-off. Then the discrete HMM score higher than the A-based HMM upper cut-off or the P-based HMM lower cut-off were assigned with a positive marker; the scores lower than the A-based HMM lower cut-off or higher than the P-based HMM upper cut-off were assigned with a negative marker (Supplemental figure S1). At the end, the proteins with a number of accumulated markers larger than 0 were predicted as anammoxosomal, and those with total number lower than 0 were predicted as cell envelope-targeted. Sequences with a final marker of 0 were considered ambiguous. For the combination of the five alignments, the final scores of the scoring matrices of every alignment were added up to give a combined prediction.

The four ungapped alignments from the N-terminal region of the proteins were then put next to each other to form ‘Frankenstein’ alignments of the signal peptides. From these alignments, one HMM was made for each set and the resulting set of two best-performing HMMs was combined in the same manner as for the other HMM sets.

For every set of the selected HMMs, 10-fold Jack-knife cross-validation was applied.

**Results**

**Signal peptide analysis**

To investigate whether the signal peptides of the two training sets A and P have any distinguishing sequence characteristics, we used an approach based on hidden Markov Models constructed using the four ungapped alignments from the signal peptides. The approach is outlined in Supplemental figure S1. Although the separation percentages attained in the separation plots of the resulting HMM pairs were often quite high (up to 100% in some large sequence windows), the models did not appear to have any separation value when tested by cross-validation, indicating that the high distinguishing percentages are caused by data overfitting rather than the presence of consistently positioned sorting signals in the sequences (Supplemental table S1). Further attempts, such as (i) combining of the four HMM pairs (one of every alignment) into a single scoring matrix (Supplemental table S1), (ii) varying the thresholds of the score matrix outcomes that guided the prediction of protein subcellular localization (data not shown) and (iii) changing the windows from which the HMMs were constructed to cover only the regions close to the aligned positions (n-region: 0 - +5; h-region: -3 - +10; c-region: -3 - +5; cleavage site: -3 - +8, yielding a cross-validation accuracy of 53.3% ) did not find any distinguishing signal peptide sequence characteristics (Supplemental table S1). The puzzled “Frankenstein” alignments of the signal peptides were built into HMMs and the above-mentioned processes were used to analyze these HMMs. However, no significant value was found to separate anammoxosomal from cell-envelope proteins according to our 10-fold cross-validation either (Supplemental table S1).

**Supplemental tables**

## Supplemental table S1 - Cross-validation results of signal peptide HMMs

| **Alignment** | **Positions** | **Cross-validation accuracies (%)** |
| --- | --- | --- |
| N-terminus | +0 to +8 | 47.4 % |
| Start H-region | -2 to +12 | 49.0% |
| Start C-region | -3 to +9 | 56.6% |
| Cleavage site | -7 to +13 | 50.0 % |
| Four alignments combined | - | 53.3% |
| C-terminus | -11 to -1 | 57.4% |

Positions of the sequence windows from which the final models were taken, and cross-validation accuracies of the set of two HMMs from this region of the alignment. Cross-validation accuracies were measured by taking the average of the percentages of correctly classified sequences for the two sets.

**Supplemental figures**

## Supplemental figure S1 - Approach of signal peptide comparison using Hidden Markov Models


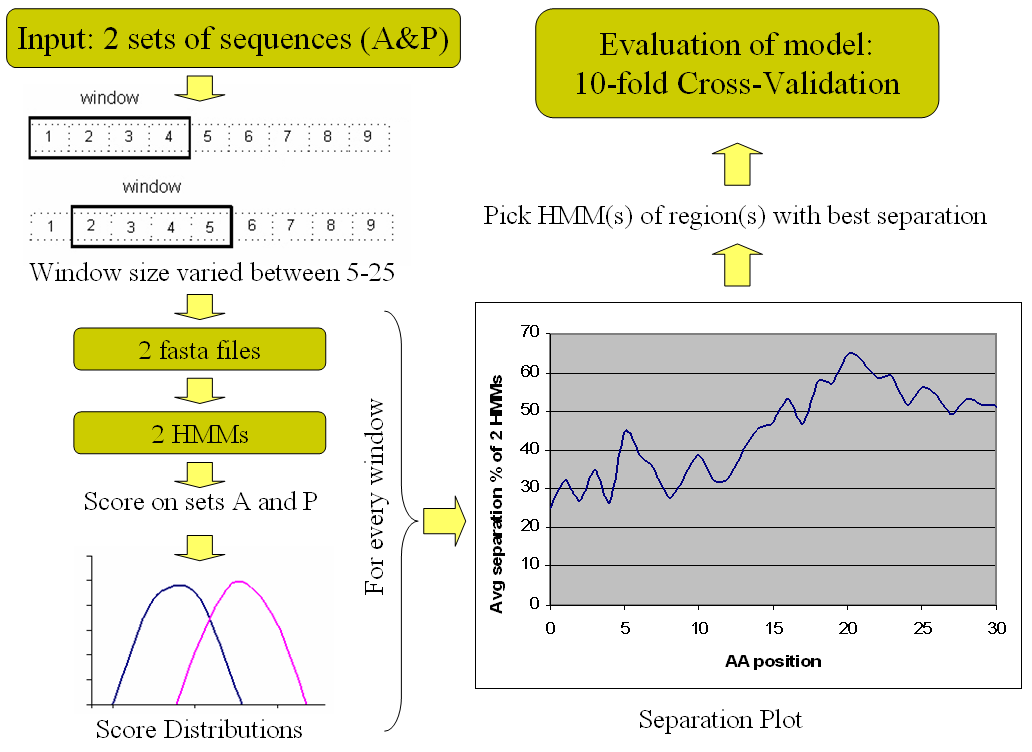


### Supplemental figure S2 - Signal peptide predictions on sets A and P by fifteen SP prediction algorithms


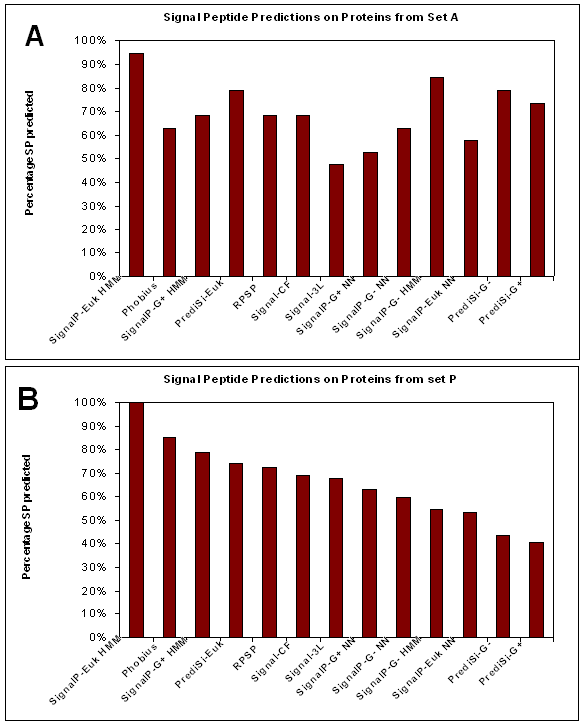


The SignalP-HMM method trained on eukaryote SPs predicts the most SPs in both set A and set P proteins. The single protein of set A that did not have a SP predicted by SignalP-Euk HMM could nonetheless manually be seen to carry a SP.

**Supplemental figure S3 - Weblogos of TMHs of K. stuttgartiensis (above) and E. coli (below)**

**
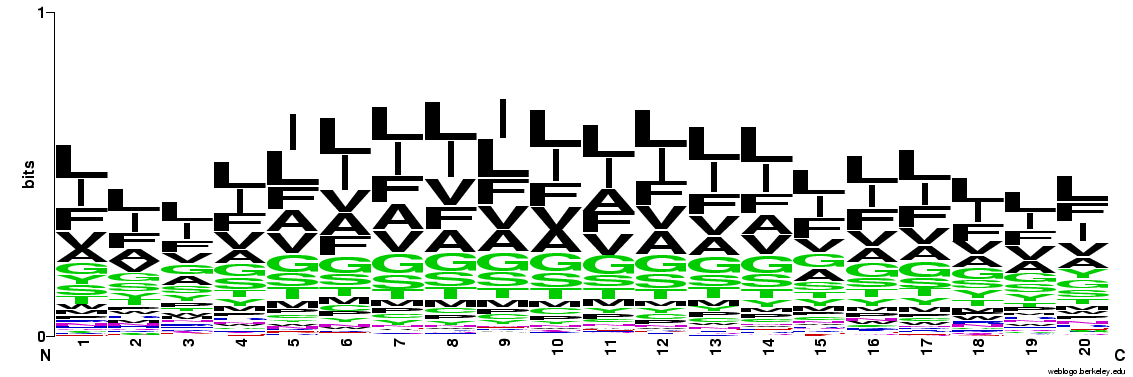

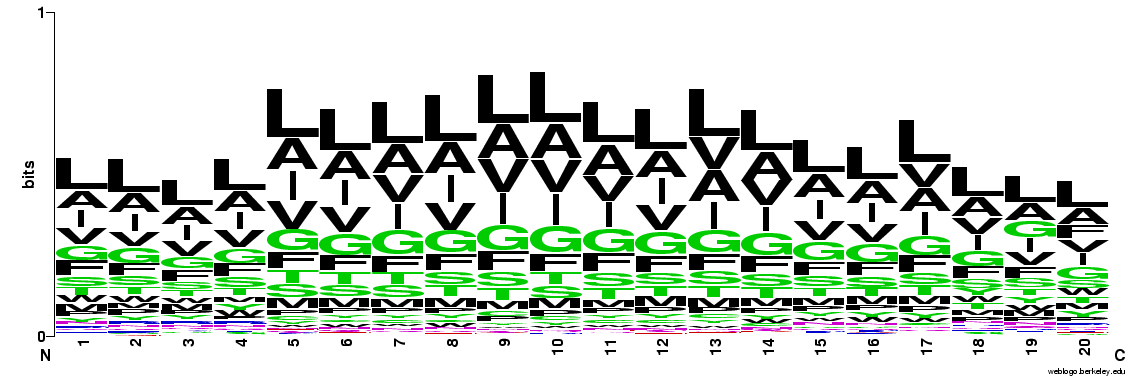
**

Weblogos were constructed from FASTA files containing all TMHs of the respective proteomes as predicted by TMHMM 2.0. Phenylalanine residues are present in significantly higher abundance in *K. stuttgartiensis* TMHs.

**References**:

1. Candiano G, Bruschi M, Musante L, Santucci L, Ghiggeri GM, Carnemolla B, Orecchia P, Zardi L, Righetti PG: **Blue silver: a very sensitive colloidal Coomassie G-250 staining for proteome analysis**. *Electrophoresis* 2004, **25**(9):1327-1333.

2. Mann M: **A shortcut to interesting human genes: peptide sequence tags, expressed-sequence tags and computers**. *Trends in Biochemical Sciences* 1996, **21**(12):494.

3. Rappsilber J, Ishihama Y, Mann M: **Stop and go extraction tips for matrix-assisted laser desorption/ionization, nanoelectrospray, and LC/MS sample pretreatment in proteomics**. *Anal Chem* 2003, **75**(3):663-670.

4. Ishihama Y, Rappsilber J, Andersen JS, Mann M: **Microcolumns with self-assembled particle frits for proteomics**. *J Chromatogr A* 2002, **979**(1-2):233-239.

5. Perkins DN, Pappin DJ, Creasy DM, Cottrell JS: **Probability-based protein identification by searching sequence databases using mass spectrometry data**. *Electrophoresis* 1999, **20**(18):3551-3567.

6. Eddy SR: **HMMER: profile HMMs for protein sequence analysis**. *Bioinformatics* 1998, **14**:755-763.
